# Dexmedetomidine administration as a possible cognitive enhancer through increasing the level of brain-derived neurotrophic factors in surgical patients: a systematic review and meta-analysis

**DOI:** 10.1186/s12871-024-02646-y

**Published:** 2024-07-26

**Authors:** Masoud Dehbozorgi, Fatemeh Fereidooni, Ramin Bozorgmehr, Javad Zebarjadi Bagherpour, Arman Shafiee, Ida Mohammadi, Mohammad Javad Amini, Niloofar Seighali, Kyana Jafarabady, Shahryar Rajai Firouzabadi, Diba Akbarzade, Razman Arabzadeh Bahri

**Affiliations:** 1 School of Medicine, RWTH Aachen University, Aachen, Germany; 2 School of Medicine, Shiraz University of Medical Sciences, Shiraz, Iran; 3 Department of Surgery, Shahid Madani Hospital, Alborz University of Medical Sciences, Karaj, Iran; 4 Student Research Committee, School of Medicine, Alborz University of Medical Sciences, Karaj, Iran; 5 School of Medicine, Shahid Beheshti University of Medical Sciences, Tehran, Iran; 6 School of Medicine, Tehran University of Medical Sciences, Tehran, Iran

**Keywords:** Surgery, Dexmedetomidine, Brain-derived neurotrophic factor, BDNF

## Abstract

**Objective:**

This meta-analysis aimed to investigate the effect of dexmedetomidine on brain-derived neurotrophic factor (BDNF) levels in individuals undergoing various medical procedures. We systematically searched electronic databases and manually identified relevant articles to assess the impact of dexmedetomidine on BDNF levels in surgical patients.

**Methods:**

A comprehensive literature search was conducted in PubMed, Scopus, Embase, and Web of Science databases with no language restrictions. Studies that examined the effects of dexmedetomidine administration on BDNF levels in surgical patients were included.

**Results:**

The overall analysis revealed a statistically significant increase in BDNF levels in individuals receiving dexmedetomidine compared to controls (Standardized Mean Difference SMD = 1.65, 95% CI: 1.02 to 2.28; I2: 89%). Subgroup analyses based on the anesthesia method (*p* < 0.01), and the type of surgery (*p* < 0.01) showed significant between-group differences (Fig. 3). The results of the sensitivity analyses indicated that individual studies did not significantly affect the overall results.

**Conclusion:**

This meta-analysis indicates that dexmedetomidine administration is associated with a significant increase in BDNF levels in individuals undergoing surgical procedures. These findings highlight the potential role of dexmedetomidine in modulating BDNF levels, which may have implications for optimizing perioperative neuroprotective strategies and improving patient outcomes.

**Supplementary Information:**

The online version contains supplementary material available at 10.1186/s12871-024-02646-y.

## Introduction

Dexmedetomidine is a highly selective α2-adrenergic agonist that has gained increasing attention in the field of anesthesia and perioperative medicine [1]. It is commonly used for its sedative and analgesic properties, offering an alternative to traditional sedatives and opioids [2]. In recent years, researchers have sought to explore the protective effects of dexmedetomidine, particularly on neurotrophic factors such as brain-derived neurotrophic factor (BDNF) in surgical patients [3].

BDNF is a neurotrophin that plays a crucial role in neuronal survival, growth, and synaptic plasticity [4]. It has been implicated in various neurological and psychiatric disorders [5], and its modulation may have implications for cognitive function and recovery in surgical patients [6]. In a previous meta-analysis, it has been reported that a decrease in BDNF levels is correlated with impaired cognition [7]. Also, Dexmedetomidine has been proposed as a modulator for BDNF levels post-operation, exerting a neuroprotective and anti-neuroinflammatory function [8].

Several studies have investigated the influence of dexmedetomidine on BDNF levels in post-surgical patients. Ge et al. [9] found that dexmedetomidine administration was associated with a significant increase in BDNF levels in patients undergoing carotid endarterectomy. In contrast to their findings is a study by Cheng et al. [10], who investigated the impact of dexmedetomidine on BDNF levels in patients undergoing total knee arthroplasty and found no significant change in BDNF levels compared to the control group.

These conflicting findings in the literature highlight the need for a systematic review and meta-analysis to provide a comprehensive evaluation of the effect of dexmedetomidine on BDNF levels in post-surgical patients. By synthesizing the available evidence, we aimed to determine the overall impact of dexmedetomidine on BDNF levels post-operation and explore potential factors that may explain the variability in the results, ultimately contributing to the advancement of perioperative care.

## Method

The present systematic review was conducted based on Preferred Reporting Items for Systematic Reviews and Meta-Analyses (PRISMA) and AMSTAR (Assessing the methodological quality of systematic reviews) guidelines and guidelines retrieved from the Cochrane Handbook for Systematic Reviews of Interventions [11].

### Search strategy

We searched databases including PubMed, Web of Science, Scopus, and Embase up to 24 May 2023, using the search string with the combination of the following keywords: (“dexmedetomidine”) AND (“brain-derived neurotrophic factor” OR “BDNF”) AND (“surgery” OR “surgical patients”) (Supplementary File). No limitation was implemented on our search results. A second search was conducted one week before the submission in order to identify any newly published studies.

### Eligibility criteria

Two reviewers, acting independently, initially evaluated the titles and abstracts of the identified articles to determine if they met the criteria for inclusion in the study, which focused on investigating the impact of dexmedetomidine administration on BDNF levels in patients who underwent any surgical procedure. Then, the full text of the initially identified studies was reviewed to make a final decision. All types of study designs, including RCTs, non-randomized controlled trials, observational studies, interrupted time series, and controlled before-and-after studies, were considered for inclusion in this study. The exclusion criteria consisted of irrelevant topics, articles that did not meet the mentioned inclusion criteria, review articles, meta-analyses, case reports, or animal studies. Disagreements between the reviewers were resolved through discussions and mutual agreement. There were no restrictions on the publication date or language of the included studies.

### Screening and data extraction

The study selection process involved two independent reviewers and was conducted in two stages. During the first stage, the reviewers assessed the relevance of all identified articles by examining their titles and abstracts in relation to the research question and inclusion criteria. In the second stage, potentially eligible studies’ full texts were obtained and thoroughly reviewed for final inclusion. Data extraction was independently carried out by the same two reviewers, utilizing a pre-defined data extraction form.

### Quality assessment

The quality of the included studies was independently assessed by the same two reviewers using appropriate tools based on the study design. For randomized controlled trials, the Cochrane Collaboration’s Risk of Bias tool was used. For observational studies, the Newcastle-Ottawa Scale was employed. Disagreements between reviewers were resolved through discussion or consultation with a third reviewer if necessary.

### Data synthesis

A meta-analysis was conducted to determine the overall impact of dexmedetomidine administration on BDNF blood levels. To account for variations in BDNF measurement methods across studies, the standardized mean difference (SMD) was employed as the effect size metric. A random effect model was utilized to address potential heterogeneity among the included studies. Heterogeneity was assessed using the I^2 statistic, where values of 25%, 50%, and 75% indicated low, moderate, and high heterogeneity, respectively. Subgroup analyses were performed based on the source of BDNF, timing of assessment, and the anesthesia method. Sensitivity analysis was conducted using the leave-one out method to evaluate the robustness of the findings. To evaluate publication bias, both visual assessments using funnel plot asymmetry and statistical analysis using Egger’s regression test were employed. A symmetric funnel plot would suggest the absence of publication bias, while Egger’s test with a p-value less than 0.05 would indicate significant publication bias.

## Results

### Study selection

**Fig. 1:**
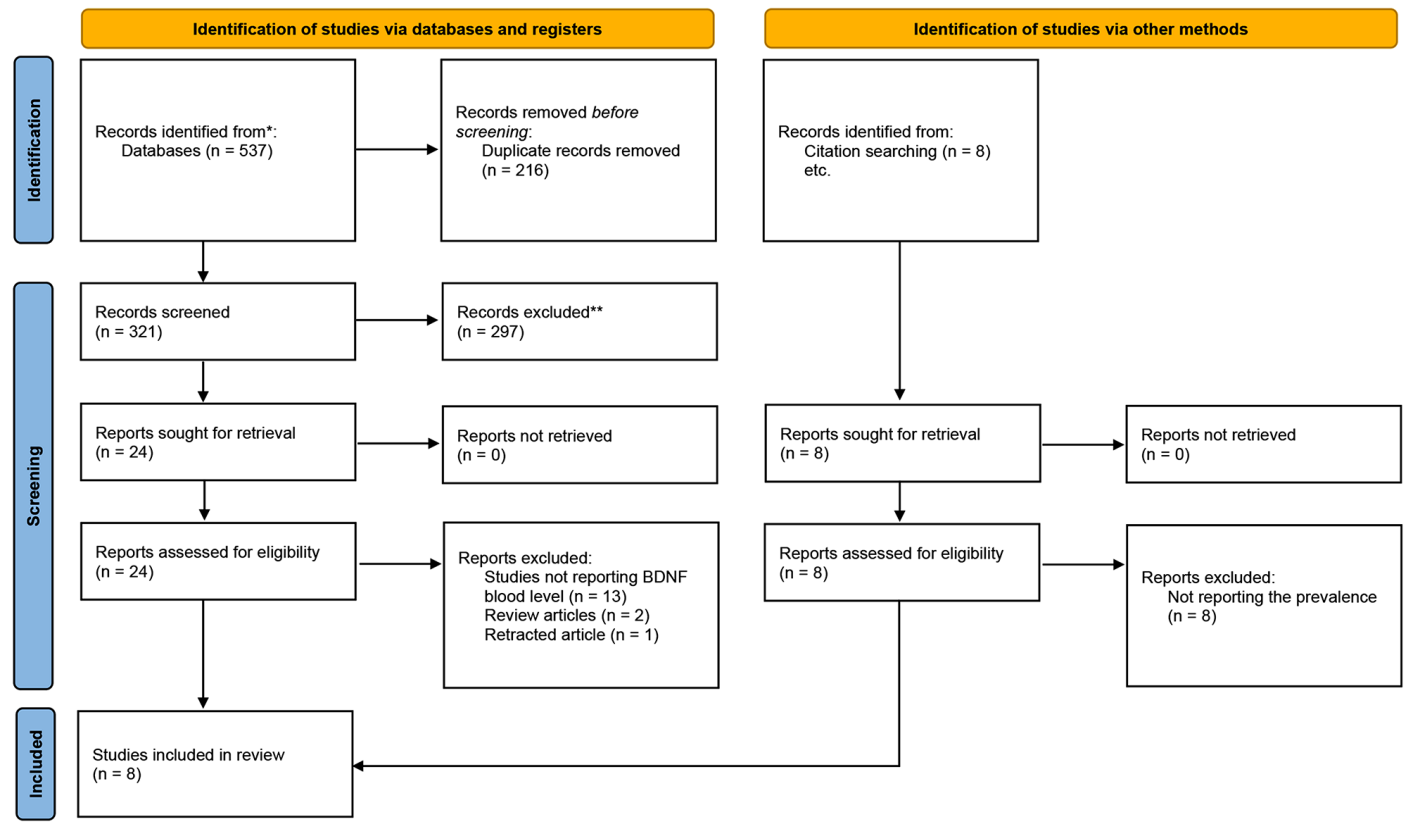
PRISMA flow diagram

A total of 537 articles were found using electronic database searches, and 8 additional articles were identified manually. After removing duplicates, 321 articles underwent screening based on title and abstract. Among these, 24 articles were selected for a full-text assessment, and finally, 8 articles met the inclusion criteria and were included in the study [3, 8, 9, 12–16] (see Fig. 1 for the PRISMA flow diagram).

### Study characteristics and quality assessment

Table 1 presents the characteristics of the included studies. The selected studies were published between 2013 and 2023. All studies were conducted in China. Among the included studies, 6 were randomized controlled trials (RCTs) [3, 8, 9, 12, 13, 16], and 2 were observational studies [14, 15]. The results of our quality assessments showed one study with a high risk of bias [12] (Supplementary File).

**Table 1:** Characteristics of included studies

Author	Year	Country	Type of study	Total participants /Total Dex/ Total control	Surgery	Type of anesthesia	Dose/administration route	Source of BDNF	Timing of assessment
Cheng, W.	2019	China	RCT	54/18/18/18	unilateral lower-extremity	spinal	Prior to tourniquet inflation, patients in the Dex group received a loading dose of Dex (0.8 µg/kg), followed by continuous infusion of Dex (0.4 µg/kg/h) until the end of surgery	serum	30 min before and at 6 h after
Cheng, W.	2023	China	RCT	36/18/18	knee arthroplasty	spinal	Before inflation of the tourniquet, patients in the Dex group received a loading dose of Dex (0.8 µg/kg over 10 min intravenously) followed by continuous infusion of Dex (0.5 µg/kg/h intravenously) until the end of surgery	serum	before anesthesia and 30 min, 6 h, and 24 h after
Ding, Q.	2020	China	Cohort	225/109/1116	surgeries with traumatic brain injuries	general	Patients of the DEX group were received intraoperative DEX 1 mg/mL	plasma	15 min before and 15 min after
Ge, Y.	2019	China	RCT	56/25/24	carotid endarterectomy	general	infusions of DEX (0.3 mg/kg loading dose, 10 min before induction of anesthesia; 0.3 microg/kg/h maintenance dose) were administered until 30 min before end of surgery	serum	20 min before, 10 min after intubation, 5 min after clamping of the carotid artery, 15 min after unclamping, 1 h postoperatively, 24 h postoperatively
Wei, X.	2019	China	Cohort	106/50/56	congenital heart disease	general	The observation group was infused with fentanyl at 10 µg/kg/h and dexmedetomidine at 1 µg/ kg/h	serum	before anesthesia, 1 h after anesthesia, immediately before incision, immediately after incision, end of the surgery 10 min after the end of surgery
Xing, C.	2021	China	RCT	110/55/55	Hip Surgery	general	the study group was injected with 0.5 mg/kg dexmedetomidine	plasma	5 min after admission, at the timeof sectioning, immediately after surgery, and 6 h after surgery
Yang, L.	2013	China	RCT	40/20/20	lumbar discectomy	general	0.6 mg/kg/h DEX was additionally administered by continuous infusion	plasma	baseline,15 min after intubation and before the surgery was started, the end of surgery, 10 min after extubation,24 h after the surgery
Zhang, J.	2018	China	RCT	58/29/29	ischemic cerebrovascular disease	general	The dexmedetomidine group was treated with intravenous administration of 1 µg/kg dexmedetomidine using a micro-injection pump before induced anesthesia	serum	3 days after operation

### Meta-analysis

**Fig. 2:**
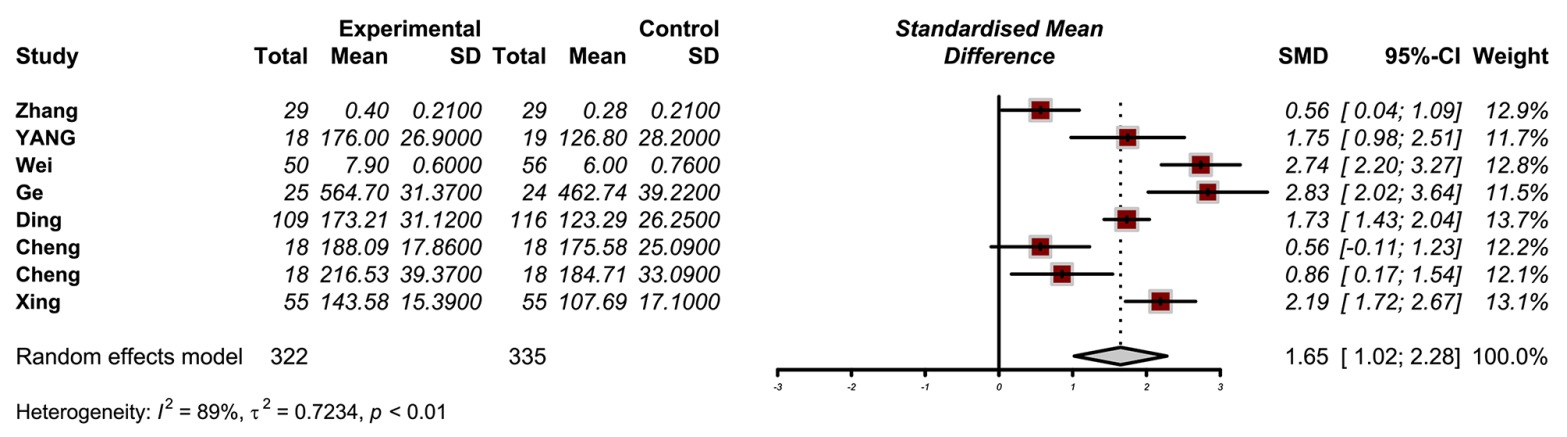
Results of meta-analysis for the level of Brain-Derived Neurotrophic Factor (BDNF) levels dexmedetomidine administration

The meta-analysis included 8 studies, providing sufficient data for pooled effect size calculation. The pooled effect size indicated a statistically significant increase in BDNF levels in individuals who received dexmedetomidine compared to controls (SMD = 1.65, 95% CI: 1.02 to 2.28; I2: 89%) (Fig. 2).

### Subgroup and sensitivity analyses

**Fig. 3:**
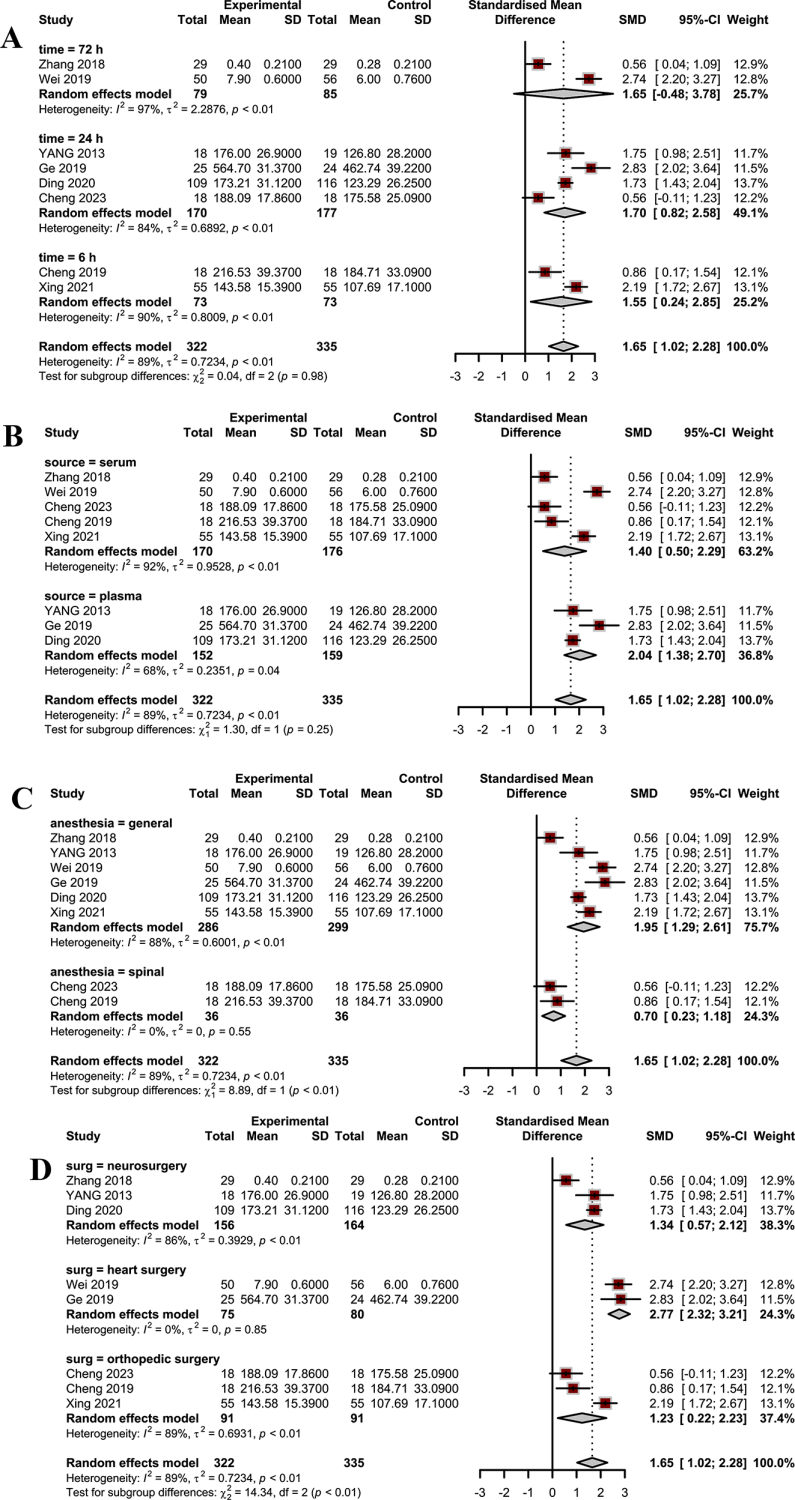
Results of subgroup analysis, (**a**) time of assessment; (**b**) source of BDNF; (**c**) type of anesthesia; and (**d**) type of surgery

**Fig. 4:**
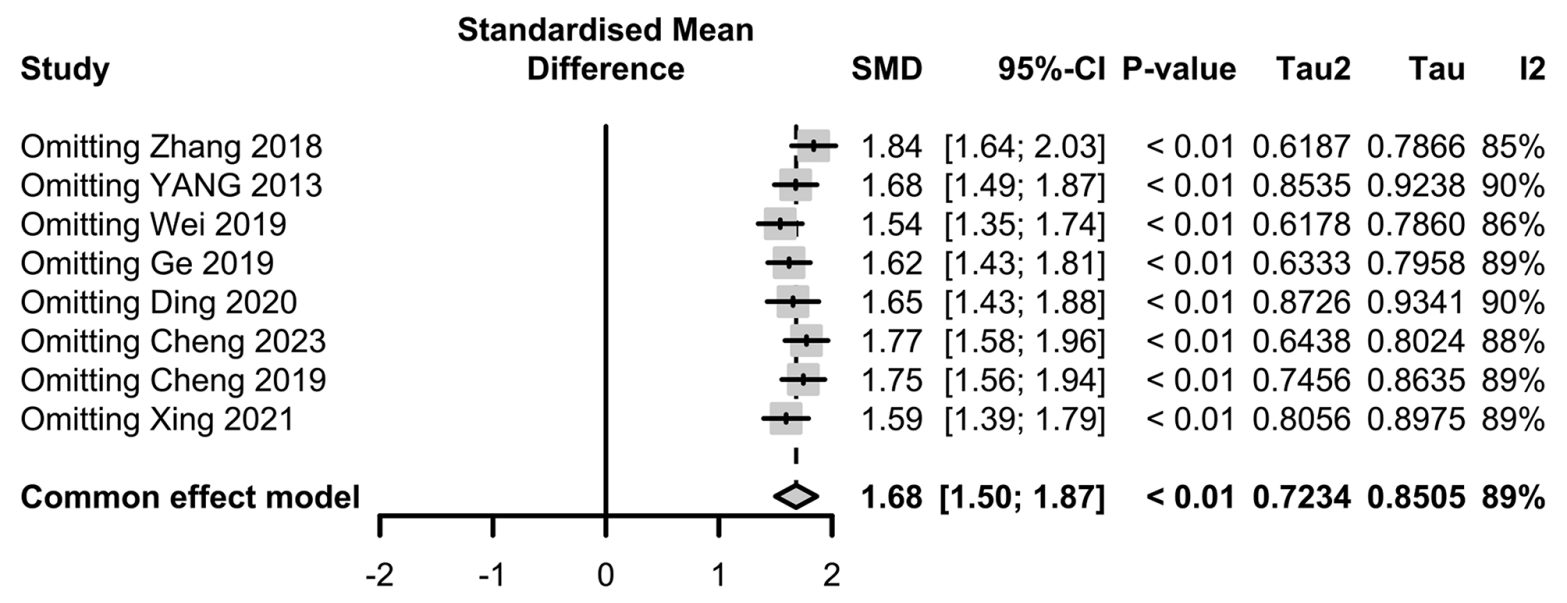
Results of sensitivity analysis

Subgroup analyses based on the anesthesia method (*p* < 0.01), and the type of surgery (*p* < 0.01) showed significant between-group differences (Fig. 3). However, there was no significant between-group difference after subgrouping studies based on source of BDNF and timing of the assessment. Sensitivity analyses were performed by excluding one study at a time to evaluate the impact of individual studies on the overall effect size. The results of the sensitivity analyses indicated that individual studies did not have a significant effect on the overall results (Fig. 4).

## Discussion

This report is the first systematic review of the literature evaluating the impact of dexmedetomidine on postoperative BDNF levels. Our data indicated that the perioperative use of dexmedetomidine caused a significantly lower reduction in BDNF levels compared to control groups. Moreover, we performed subgroup analysis based on outcomes and characteristics that had sufficient reported data among the studies for conducting the analysis, including BDNF sources, timing of assessment, and anesthesia method. The subgroup analysis revealed that the elapsed time between surgery and BDNF level evaluation, the source of BDNF, and the type of anesthesia (general anesthesia or spinal anesthesia) were all significant factors influencing the level of BDNF reduction. Our results are in concordance with results from preclinical studies demonstrating the neuroprotective effects of dexmedetomidine by increasing the expression of BDNF [17, 18].

A common neurological complication of surgery, postoperative cognitive dysfunction (POCD) is manifested clinically as declined learning, memory, concentration, and execution ability, hence negatively influencing patients’ lives [19]. Although the mechanisms of its pathogenesis remain largely unknown, a reduction in BDNF levels has recently been implicated as a pathway for POCD [20, 21]. Numerous animal and human studies have found that central BDNF levels decline post-operation, and have linked this reduction with inhibited neurogenesis [22], resulting in unfavorable postoperative cognitive outcomes [23]. This downregulation has been attributed to the preoperative general anesthetic agents [24] as well as the inflammation caused by the surgery itself [25]. Our study has demonstrated that the postoperative decrease in BDNF can be at least partly remedied by the use of dexmedetomidine, potentially alleviating cognitive dysfunction. In fact, four of the studies included have demonstrated that the use of dexmedetomidine can enhance postoperative neurocognitive outcomes compared to control groups [9, 12, 14, 26], with one directly associating cognitive function with BDNF levels [26]. It is also worth mentioning that our findings were in accordance with a multitude of systematic reviews examining the effects of dexmedetomidine on postoperative cognitive outcomes, with all concluding that dexmedetomidine has a favorable impact on such outcomes [27–29].

In one of our sub-group analyses, we compared BDNF levels in the blood after surgery based on the time of evaluation. Although the number of studies for each time-point is too small to infer any meaningful interpretations, we did find that BDNF levels 30 min after surgery do not seem to differ significantly between the dexmedetomidine group and the control group, and BDNF begins to fall after at least 6 h following surgery in the control groups, a decline that’s inhibited by dexmedetomidine administration. Yet, this inhibition appears to last only three days post-operation as BDNF levels after this time-point do not differ significantly between the two groups. This 66-hour-long time period is of special importance as complications such as postoperative delirium commonly develop during this phase [30], and postoperative cognitive dysfunction may follow [31]. BDNF has previously been proposed as a biomarker for postoperative delirium and postoperative cognitive dysfunction [31].

In another of our sub-group analyses, we compared the studies that evaluated serum BDNF levels post-operation with those that evaluated plasma BDNF. Plasma BDNF is believed to be more representative of central BDNF levels [32] in comparison to serum BDNF, and in our analysis, we found both to be significantly higher in the dexmedetomidine-administered group in comparison to controls. Although more studies measuring BDNF in each are required, based on the data we had it appears that dexmedetomidine is more effective at increasing plasma BDNF in comparison to serum BDNF. This finding is in accordance with preclinical studies observing dexmedetomidine penetrating the blood-brain barrier, potentially increasing central BDNF levels, which in turn increase plasma BDNF levels [1].

Ozer et al. [24] have shown that general anesthetics decrease BDNF levels following surgery, a decline not evident after administering spinal anesthetics. Therefore, we divided the studies based on the anesthetics they used as a control, and we noticed that dexmedetomidine, although more effective in elevating BDNF levels in both groups, was more effective in studies using general anesthetics than spinal ones. Although more research is required comparing dexmedetomidine with each type of anesthetic, it appears to be a viable alternative to other adjuvant anesthetics in general anesthesia, especially in older patients who are at a heightened risk of cognitive impairments [33].

This review has several potential limitations. First, five of the included studies had a sample size of less than one hundred patients, which are described as small studies. Therefore, our study may be subject to small study effect bias. Additionally, the duration and dosage of dexmedetomidine administration differed across studies and the influence of these factors on our results cannot be ruled out. In addition, the included studies performed different types of surgeries and the possible correlation of BDNF and type of the surgery after administration of dexmedetomidine needs further evaluation. Moreover, all studies included were carried out in China, not only increasing risk of publication bias, but also decreasing our ability to generalize these results to other ethnicities. It is also notable that we used circulating BDNF as a proxy for drawing conclusions about central BDNF. While this assumption is not baseless [32], central BDNF is surely the superior measure when discussing neuroprotection. Also, we performed meta-analysis on studies with different study designs, including RCTs and observational studies, which have different patient randomization processes, which may have a significant impact on the heterogeneity of the included studies.

## Conclusion

This review indicated that the use of dexmedetomidine significantly lowers the reduction of circulating BDNF that usually follows anesthesia and surgery. This finding was more pronounced in plasma, in patients receiving general anesthesia, and in the relatively short term. Our results support the notion that dexmedetomidine exerts neuroprotective effects in patients undergoing surgery, although further research is needed to bolster these findings by examining the effects of dexmedetomidine on central BDNF levels.

### Electronic supplementary material

Below is the link to the electronic supplementary material.

Supplementary Material 1

## Data Availability

All data has been presented in the manuscript.
